# Overexpression of *Acyl-CoA-Binding Protein 1* (*ChACBP1*) From Saline-Alkali-Tolerant *Chlorella* sp. Enhances Stress Tolerance in *Arabidopsis*

**DOI:** 10.3389/fpls.2018.01772

**Published:** 2018-11-28

**Authors:** Kun Qiao, Min Wang, Tetsuo Takano, Shenkui Liu

**Affiliations:** ^1^The State Key Laboratory of Subtropical Silviculture, Zhejiang Agriculture and Forestry University, Lin’an, China; ^2^Shenzhen Key Laboratory of Marine Bioresource & Eco-Environmental Science, Guangdong Engineering Research Center for Marine Algal Biotechnology, College of Life Science and Oceanography, Shenzhen University, Shenzhen, China; ^3^Key Laboratory of Saline-alkali Vegetation Ecology Restoration in Oil Field (SAVER), Ministry of Education, Alkali Soil Natural Environmental Science Center (ASNESC), Northeast Forestry University, Harbin, China; ^4^Asian Natural Environment Science Center (ANESC), University of Tokyo, Tokyo, Japan

**Keywords:** saline-alkali-tolerant (SAT) *Chlorella*, acyl-CoA-binding protein 1, gene expression, stress tolerance, *in vitro* filter-binding, phosphatidylcholine

## Abstract

A large proportion of the world’s arable land is saline-alkali land, and this is becoming an urgent environmental problem for agriculture. One approach to address this problem is to develop new varieties of stress-resistant plants through genetic engineering. The algae (*Chlorella* *sp.*) JB6, which was previously isolated from saline-alkali land, was found to exhibit strong NaHCO_3_ tolerance. Here, we explored saline-alkali-tolerance genes in this alga that might be useful for producing abiotic stress-resistant transgenic plants. We identified a gene encoding acyl-CoA-binding protein 1 (ACBP1) from JB6 by screening a full-length cDNA library in yeast under NaHCO_3_ stress. Northern blot analyses showed that the *ChACBP1* mRNA levels were significantly up-regulated under abiotic stresses such as salinity, oxidation, heavy metals, and low temperature stresses. The recombinant ChACBP1 protein was found to bind phosphatidylcholine *in vitro*. Green fluorescent protein-labeled ChACBP1 was localized to the cytosol. Overexpression of *ChACBP1* in yeast and *Arabidopsis* increased their resistance to high salinity, oxidation, heavy metals, and low temperature stresses. These results suggested that ChACBP1 may mediate plant abiotic stress adaptation through phospholipid metabolism. Thus, ChACBP1 may be useful to genetically improve the tolerance of plants to saline-alkali soil.

## Introduction

Saline-alkali soil can contain different types of salts (e.g., Na_2_SO_4_, NaCl, NaHCO_3_). Neutral salts (NaCl and Na_2_SO_4_) and alkaline salts (NaHCO_3_ and Na_2_CO_3_) are highly destructive to plants (Guo et al., 2016; Song et al., 2017). Soils rich in NaHCO_3_ and Na_2_CO_3_ are characterized by a high pH, a high exchangeable sodium percentage, a very low water-permeability range, and saturated hydraulic conductivity (Shi et al., 2012; Qiao et al., 2015). Such environments, which are known as “carbonate stressed” environments, are usually barren with only a few scattered plants (Qiao et al., 2015). Carbonate stress threatens the development of agriculture and livestock husbandry. Carbonate stress impairs plants in several ways by imposing ionic stress, drought stress, and oxidative stress, resulting in nutritional deficiency, metabolic disorders, and membrane damage. These changes affect plant growth and development and agricultural yield. The induction of proteins associated with lipid metabolism and lipid signaling has been proposed to be an important factor in plants’ acclimation to stress conditions (Xiao and Chye, 2011).

In plants, lipids are one of the main components of biological membranes and play important roles in diverse biological processes, such as the provision of energy for cell metabolism and the maintenance of organelle integrity and composition. During lipid metabolism in plant cells, lipids, and their derivatives are transferred within or across subcellular compartments with the aid of lipid-transfer proteins or acyl-CoA-binding proteins (ACBPs) (Xiao and Chye, 2009). *Arabidopsis thaliana* has six ACBPs (AtACBP1–AtACBP6) with a conserved acyl-CoA-binding domain that range in size from 10 to 73 kDa (Xiao and Chye, 2011). *In vitro*, ACBPs bind phospholipids such as phosphatidylcholine (PC) (Chen et al., 2008), phosphatidic acid (PA) (Du et al., 2010), phosphatidylethanolamine (PE) (Xiao et al., 2011), and lysophosphatidylcholine (lysoPC) (Gao et al., 2010). Plant ACBPs participate in several biological processes, including early embryogenesis (Chen et al., 2010) and leaf senescence (Xiao et al., 2011), and in responses to heavy metals (Xiao et al., 2008; Gao et al., 2010; Du et al., 2015), drought (Du et al., 2013), and freezing temperatures (Chen et al., 2008). A small 10-kDa ACBP has been identified in several plants including *Brassica napus* (Hills et al., 1994; Brown et al., 1998), *A. thaliana* (Engeseth et al., 1996), and agricultural crops including cotton (*Gossypium hirsutum*) (Reddy et al., 1996) and rice (*Oryza sativa*) (Suzui et al., 2006). The small ACBP has a housekeeping function in the intracellular transport of acyl-CoAs (Mandrupl et al., 1992) and has been well-characterized in many eukaryotic organisms (Metzner et al., 2000). *In* *vitro*, the small ACBPs of *A. thaliana* (AtACBP6) and *Helianthus annuus* (HaACBP) have been observed to bind only PCs (Chen et al., 2008; Aznar-Moreno et al., 2016). In addition to its involvement in lipid metabolism, AtACBP6 has also been reported to enhance freezing tolerance and affect jasmonate composition (Chen et al., 2008; Ye et al., 2016). Although several studies have examined the role of ACBP in abiotic stresses in higher plants, little is known about its role in algae.

Previously, we found a species of *Chlorella*, JB6, in the alkaline-saline soil of northeastern China. This alga was found to show extremely high tolerance to NaHCO_3_ and NaCl (Wang et al., 2011; Qiao et al., 2015). Therefore, it is of great significance as a genetic resource to generate transgenic stress-tolerant plants, and for studies on the responses to abiotic stresses.

In this study, we identified a gene encoding a small ACBP (*ChACBP*) from *Chlorella* (JB6) by screening its full-length cDNA library expressed in yeast under NaHCO_3_ stress. We investigated *ChACBP* expression in response to several abiotic stresses (high salinity, oxidation, heavy metals, and low temperature). Transgenic yeast and *Arabidopsis* overexpressing *ChACBP* showed enhanced tolerance to salinity, oxidation, heavy metals, and low-temperature stresses. Filter-binding assays revealed that ChACBP interacted with lipids. These results have clarified the molecular mechanism of ChACBP and indicate that the gene encoding this protein may be useful for generating transgenic plants resistant to saline-alkali soil.

## Materials and Methods

### Materials

The saline-alkali-tolerant (SAT) microalga *Chlorella* JB6 was isolated and screened from extremely alkaline-saline soil (pH > 10) from the Songnen Plain (46°27′N, 125°22′E, Heilongjiang Province, China) (Wang et al., 2011; Qiao et al., 2015) and was cultivated in liquid Bold’s basal medium (BBM) (Bold and Wynne, 1978). The *Chlorella* cells were cultured at 23 ± 1°C under white light (40 μmol photons m^-1^ s^-1^) and an 16 h light/8 h dark photoperiod. Total RNA was isolated from *Chlorella* after 100 mM NaHCO_3_ treatment. An In-Fusion^®^SMARTer^®^Directional cDNA Library Construction Kit (Cat. 634933, Clontech, Palo Alto, CA, United States) was used to construct the full-length cDNA library. Mixed plasmids were expressed in *Saccharomyces cerevisiae* InVSCI using the PEG/LiAC method. The full-length cDNA yeast library was provided by the Alkali Soil Natural Environmental Science Center (ASNESC) at Northeast Forestry University (Harbin, China).

### Gene Cloning and Analysis

The *Chlorella* acyl-CoA-binding protein gene (*ChACBP*) was amplified from a cDNA library expressed in yeast under 25 mM NaHCO_3_ stress. The full-length sequence of *ChACBP* was obtained from the NCBI website^1^. The open reading frame (ORF) and the predicted protein sequence were determined and analyzed using DNASTAR Lasergene v7.1 software^2^. Multiple sequences were aligned with Genedoc 3.0. A phylogenetic tree was constructed using the neighbor-joining (NJ) method with Mega 3.1 software.

### Yeast Transformation and Stress Tolerance Assays

The cDNA fragment of *ChACBP* was ligated into the pYES2 vector (Invitrogen, Carlsbad, CA, United States) digested with *Bam*HI and *Not*I to construct the plasmid pYES2-*ChACBP*. This construct and the pYES2 empty vector (control) were transformed into *S. cerevisiae* (InVSCI) using the PEG/LiAC method. To check the stress tolerance of transgenic lines, yeast cells expressing the pYES2-*ChACBP* vectors were incubated in liquid uracil-minus medium overnight at 30°C (FunGenome, Beijing, China), then adjusted to an OD_600_ of 0.5, and further diluted to 10^-1^, 10^-2^, 10^-3^, and 10^-4^ with sterile H_2_O. Then, 4.5 μL of each dilution series was spotted onto solid medium (yeast extract 20 g/L; peptone 20 g/L; galactose 20%) supplemented with 35 mM NaHCO_3_, 0.8 M NaCl, 3 mM H_2_O_2_, or 8 mM CuCl_2_. The cultures were grown for 3–7 days at 30°C. For the low temperature treatment, diluted yeast cells spotted onto solid medium were cultured for 20 days at 10°C.

### Purification of Recombinant His-Tagged ChACBP for Filter-Binding Assays

To construct the pQE30-*ChACBP* expression plasmid, the *ChACBP* cDNA fragment was amplified from the plasmid pYES2-*ChACBP* using the following primers: forward: 5′-GGAT CCATGGGCCTCAAGGAAGAC-3′ (*Bam*HI site underlined); and reverse 5′-GTCGACTCAAGCGTACTTCGCCTTC-3′ (*Sal*I site underlined). The amplified fragment was inserted into the pQE30 vector (Qiagen, Hilden, Germany), which was then transformed into *Escherichia coli* M15 cells. The ChACBP protein was induced, ultrasonicated, and loaded onto Ni–NTA Sefinose (Qiagen). The bound fusion protein was eluted using 250 mM imidazole (Sigma-Aldrich, St. Louis, MO, United States). Protein samples were denatured at 100°C for 8 min and then separated by SDS-PAGE. After electrophoresis, the protein products were transferred to a nylon membrane (Amersham, Little Chalfont, United Kingdom) using a Hoefer^TM^ TE 70 semi-dry transfer unit (Amersham) for 90 min, then blocked for 1 h in 1% blocking buffer (QIAexpress Anti-His HRP Conjugate kit), probed with Penta anti-(His)_6_ horseradish peroxidase (HRP)-conjugate antibody (Qiagen) for 90 min at 25°C, and then detected with an ECL Western Blotting Substrate kit (Abcam, Cambridge, United Kingdom) using a Luminescent Image Analyzer LAS-4000 (Fujifilm, Tokyo, Japan). Binding of ChACBP to various lipids on a nitrocellulose filter membrane was detected as described previously with minor modifications (Chen et al., 2008). Briefly, various lipids were spotted onto the membrane and incubated overnight at 25°C in the dark. The lipids PC, PA, 16:0-PC, 18:0-PC, and 18:1-PC (total acyl carbon: double bonds) were purchased from Sigma; and phosphatidylglycerol (PG), phosphatidyl-serine (PS), PE, and 1,2-dimyristoyl-sn-glycero-3-phosphocholine (DMPC) were purchased from Echelon Biosciences (Salt Lake City, UT, United States). The lipid-bound membrane was blocked with 1% (w/v) nonfat milk for 1 h, and then with 2 μg/mL ChACBP protein for 2 h. The membrane was incubated with the Penta anti-(His)_6_ HRP antibody for 2.5 h at 25°C, detected with ECL reagent, and analyzed using the LAS-4000 imager.

### Expression Analysis of *ChACBP* Under Stress Treatments

To check the expression level of *ChACBP* under various abiotic stresses including high salinity, oxidation, and heavy metal stresses, *Chlorella* cells were grown in liquid medium supplemented with 200 mM NaHCO_3_, 200 mM NaCl, 2 mM H_2_O_2_, or 100 μM CuCl_2_, respectively. Low temperature stress was applied by incubating the microalga at 4°C. Samples were collected at 0, 3, 6, 12, 24, and 48 h for analysis. The concentration gradients in the stress treatments were as follows: 0, 50, 100, 200, 300, and 400 mM NaHCO_3_; 0, 50, 100, 150, 200, and 300 mM NaCl; 0, 1, 2, 3, 4, and 5 mM H_2_O_2_; 0, 50, 100, 200, 300, and 500 μM CuCl_2_; and temperatures of 24°C, 18°C, 16°C, 14°C, 10°C, and 4°C. Samples were collected at 6 h of these treatments, and ground in liquid nitrogen with a mortar and pestle.

Total RNA was extracted using RNAiso plus (TaKaRa, Kyoto, Japan). Northern blot analysis was performed using the Digoxigenin Nucleic Acid Detection kit (Roche, Basel, Switzerland). Total RNA (3 μg) was separated on a 1.5% agarose gel and transferred to a Hybond-N membrane (Amersham). The *ChACBP* cDNA probes were obtained using the PCR Digoxigenin Probe Synthesis kit according to the manufacturer’s instructions (Roche). Hybridization was performed according to standard procedures recommended by the manufacturer (Roche), and then detected using the CDP Star system with the LAS-4000 imager.

### Localization of ChACBP Protein in Plant Cells

To construct the expression plasmid pBI121-*ChACBP*-GFP, *ChACBP* was amplified from the pYES2-*ChACBP* plasmid by PCR using the primers ChACBP-GFP-F (5′-GGATCCATGGGC CTCAAGGAAGACTTTG-3′; *Bam*HI site underlined) and ChACBP-GFP-R (5′-GGTACCGGAGCGTACTTCGCCTTCAG CG-3′; *Kpn*I site underlined). The PCR product was introduced into the pEGFP plasmid (Clontech) at the *Bam*H1 and *Kpn*I sites. Then, *ChACBP*-pEGFP was digested with *Bam*HI and *Not*I and ligated into the pYES2 vector (Invitrogen). The plasmid pYES2-*ChACBP*-GFP was inserted into the pBI121 vector via the *Bam*HI and *Xho*I digestion sites to construct the plasmid pBI121-*ChACBP-*GFP. *Agrobacterium tumefaciens* strain EHA105 was transformed by electroporation with either pBI121-GFP or pBI121-*ChACBP*-GFP. Transgenic *A. tumefaciens* cells were transformed into wild-type (WT) *Arabidopsis* (Columbia-0) by the floral dip method (Clough and Bent, 1998). Transgenic lines overexpressing pBI121-*ChACBP-*GFP were selected on 1/2 MS medium containing kanamycin (40 μg/mL) and confirmed by northern blot analysis using a *ChACBP* cDNA probe. *Arabidopsis* protoplasts were prepared by Sheen’s method (Sheen, 2002). All GFP signals were detected using a laser-scanning confocal imaging system (Olympus, Tokyo, Japan).

### Generation of *ChACBP*-Overexpressing Plants

To construct the plasmid pBI121-*ChACBP*, the full-length *ChACBP* was amplified from pYES2-*ChACBP* by PCR using the primers pBI-F (5′-GGATCCATGG GCCTCAAGGAAGACTTTG-3′; *Bam*HI site underlined) and pBI-R (5′-GTCGACTCAAGCGTACTTCGCCTTCAG-3′; *Sal*I site underlined). The PCR product was ligated into the pBI121 plasmid (Clontech) via the *Bam*HI and *Sal*I sites. The construct was amplified in *E. coli* and then used to transform *A. tumefaciens*. The positive transformants were confirmed by Northern blot and Southern blot analyses using a *ChACBP* probe. Genomic DNA was extracted from the leaves of 12-day-old seedlings (WT, OX-2, OX-3, and OX-4) using the CTAB method. The genomic DNA was digested with *Bam*HI, and then separated by electrophoresis on a 1.5% agarose gel and blotted onto a nylon membrane. Southern blotting was conducted with the CDP Star system and analyzed using the LAS-4000 imager.

### Stress Tolerance of *ChACBP* Transgenic Plants

The WT and transgenic *Arabidopsis* seeds were sterilized as described by Zhang et al. (2008). To measure root growth and fresh weight, the WT and transgenic *Arabidopsis* seeds were sown on 1/2 MS solid agar plates supplemented with 2 and 3 mM NaHCO_3_, 125, and 150 mM NaCl, 2, and 3 mM H_2_O_2_, or 50 and 80 μM CuCl_2_. The seedlings were grown for 7–14 days at 23 ± 1°C under a 8-h light/16-h dark photoperiod. For the low temperature stress, the plates were incubated at 16°C and 14°C for 30 days. Root length and fresh weight were measured, and the mean ± standard error were calculated from three independent experiments. Statistical analysis was performed using SPSS 13.0 software. An LSD *t*-test was used to compare the mean values among different groups (*P* < 0.05).

### Electrolyte Leakage

Two-week-old seedlings of WT and three overexpressing lines were grown under NaHCO_3_ (0, 1, 3, 5, 7, and 10 mM), NaCl (0, 100, 125, 150, 175, and 200 mM), H_2_O_2_ (0, 1, 3, 5, 7, and 9 mM), CuCl_2_ (0, 20, 50, 80, 100, and 120 μM), and temperature (24°C, 18°C, 16°C, 14°C, 12°C, and 10°C) stress conditions for 24 h. Seedling samples were immersed in deionized water. The solution was gently agitated at 25°C for 1 h before measuring its conductivity. The total ion content was determined after heating samples to 100°C for 10 min and then cooling to 25°C. Ionic leakage was detected using a conductivity meter (DDS-307, Leici Co., Ltd., Shanghai, China).

## Results

### Identification and Characterization of *ChACBP*

**FIGURE 1:**
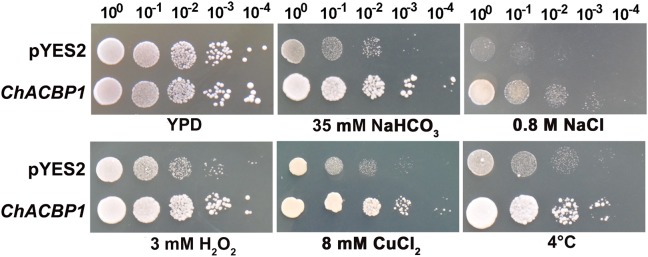
Tolerance analysis of yeast expressing *ChACBP1* to various abiotic stresses. Yeast cells containing either pYES2 empty vector or pYES2-*ChACBP1* were incubated as described in Materials and Methods. Serial dilutions were spotted onto yeast extract/peptone/galactose solid plates supplemented with NaHCO_3_, NaCl, H_2_O_2_, or CuCl_2_ at indicated concentrations. Solid yeast extract/peptone/glucose media (YPD) was the control. Growth was monitored for 3–7 days at 30°C. In the cold stress treatment, yeast cells were grown for 15 days at 10°C.

The SAT *Chlorella* was isolated from extremely alkaline-saline soil (pH > 10). The *Chlorella* cells were previously shown to tolerate 1 M NaHCO_3_ and 600 mM NaCl (Qiao et al., 2015). A cDNA library of *Chlorella* was expressed and screened in the InVSC1 strain of *S. cerevisiae* to identify potential genes involved in the strong stress tolerance of this microalga. Potential overexpression candidates were further verified under high NaHCO_3_ conditions (Supplementary Figure S1), and genes were isolated and sequenced (Supplementary Figure S2). The *acyl-CoA-binding protein* gene (*ACBP*) was isolated from SAT *Chlorella* for the first time (white arrow), and was named *ChACBP1*. Yeast tolerance analyses showed that *ChACBP-*expressing transformants grew better than the controls (YPD) under 35 mM NaHCO_3_, 0.8 M NaCl, 3 mM H_2_O_2_, 8 mM CuCl_2_, and at 10°C (Figure 1).

**FIGURE 2:**
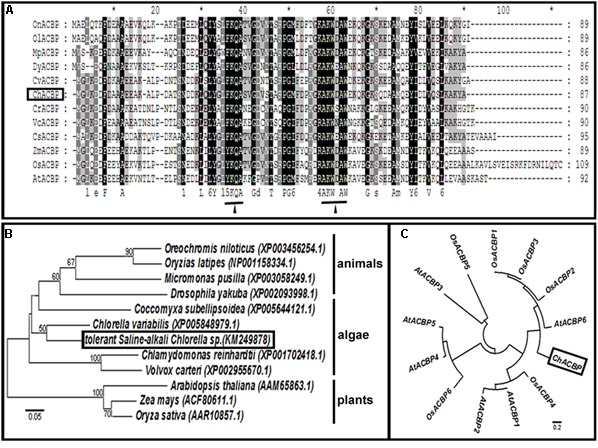
Phylogenetic and sequence analyses of acyl-CoA-binding protein 1 (ChACBP1) from saline-alkali-tolerant *Chlorella*. **(A)** Sequence alignment of ACB domain from ChACBP1 with those of other species. YKQA and KWDAW motifs are underlined; capital letters indicate identical residues in all ACBPs; small letters indicate residues identical in most ACBPs (arrowheads). Black background indicates conserved residues. On, *Oreochromis niloticus*; Ol, *Oryzias latipes*; Mp, *Micromonas pusilla*; Dy, *Drosophila yakuba*; Cv, *Chlorella variabilis*; Ch, Saline-alkali-tolerant *Chlorella* sp.; Cr, *Chlamydomonas reinhardtii*; Vc, *Volvox carteri*; Cs, *Coccomyxa subellipsoidea*; Zm, *Zea mays*; Os, *Oryza sativa*; At, *Arabidopsis thaliana*; ChACBP1 is indicated in boldface in box. **(B)** Neighbor-joining (NJ) phylogenetic relationships among acyl-CoA-binding protein (ChACBP) from saline-alkali-tolerant *Chlorella* and various ACBPs from other species. Bootstrap values were calculated 1,000 times; values < 50% are not shown. Saline-alkali-tolerant *Chlorella* is indicated in boldface in box. GenBank accession numbers are as follows: OnACBP (XP_003456254.1), OlACBP (NP_001158334.1), MpACBP (XP_003058249.1), DyACBP1 (XP_002093998.1), CvACBP1 (XP_005848979.1), ChACBP (KM249878), CrACBP (XP_001702418.1), VcACBP (XP_002955670.1), CsACBP (XP_005644121.1), ZmACBP (ACF80611.1), OsACBP (AAR10857.1), and AtACBP (AAM65863.1). **(C)** Neighbor-joining phylogenetic relationships among ChACBP and members of ACBP families in *Arabidopsis thaliana* and rice. Box indicates ChACBP.

Analysis of the *ChACBP1* sequence indicated that the ORF was 264 nucleotides long, and encoded a predicted protein of 87 amino acids. The cDNA sequence was found to contain a 57-bp 5’-untranslated region (UTR) and a 204-bp 3’-UTR. The amino acid sequence of ChACBP1 was found to share high identity with previously published ACBP sequences from other algae, plants, and animals. A comparison of the ACB domains of these ACBPs suggested conservation of the YKQA and KWDAW motifs (Figure 2A). In a neighbor-joining (NJ) tree of the ACBPs, ChACBP1 was closer to the ACBP of *Chlorella variabilis* (XP_005848979.1) than to the ACBPs of other species. In the tree, the SAT *Chlorella* first clustered with unicellular microalgae, then with plants and animals (Figure 2B), in agreement with the expected taxonomy. In a circular NJ tree, ChACBP1 was closer to AtACBP6 and OsACBP4 than to the ACBPs of *Arabidopsis* and rice (Figure 2C).

### ChACBP Interacted With Phospholipid PC *in vitro*

**FIGURE 3:**
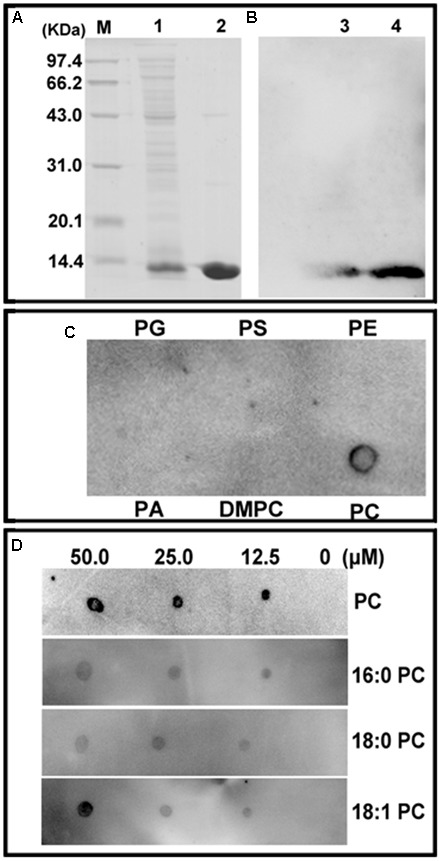
Purification of (His)_6_-ChACBP1 recombinant protein and its interaction with phosphatidylcholine (PC). **(A)** Purification of ChACBP1 protein: 9.5-kDa ChACBP1 protein was purified by Ni–NTA agarose and analyzed by SDS-PAGE. M, Marker; Lane 1, pQE30-ChACBP bacterial lysate; lane 2, purified ChACBP1 protein. **(B)** Western blot analysis of ChACBP1: protein was transferred to nylon membrane, then probed with HRP-conjugated anti-(His)_6_ antibodies. Lanes 3–4, immunoblot of (His)_6_-ChACBP1 fusion protein. **(C)** Binding of (His)_6_-ChACBP1 and lipid on filter membrane. Lipids (50.0 μM of PG, PS, PE, PA, DMPC, and PC) were spotted onto nitrocellulose and incubated with ChACBP1 protein. Binding of ChACBP1 to lipids was detected by ECL reagent with HRP-conjugated anti-(His)_6_ antibodies. **(D)** Binding of (His)_6_-ChACBP1 to various PC acyl species. Different concentrations (0, 10, 25.0, and 50.0 μM) of 16:0 PC, 18:0 PC, and 18:1 PC were spotted onto nitrocellulose and incubated with ChACBP1. Binding of ChACBP1 to lipids was detected by ECL reagent.

The *E. coli* M15 cells transformed with the pQE30-*ChACBP1* plasmid produced His-ChACBP1 fusion protein (9.5 kDa) (Figure 3A, lane 1). Elution of the His-ChACBP1 fusion protein with 250 mM imidazole gave a pure protein of the expected size (Figure 3A, lane 2). Western blot analysis showed that the purified ChACBP1 protein was not degraded (Figure 3B). To detect the interaction between ChACBP1 and phospholipids (PC, PA, PS, PG, PE, DMPC), which influences abiotic stress tolerance, we tested the binding of purified ChACBP1 to various lipids. In the binding assays, ChACBP1 specifically bound PC, but not other lipids (Figure 3C). Further analyses showed that ChACBP1 was able to bind several species of PC (16:0-PC, 18:0-PC, and 18:1-PC) (Figure 3D).

### Induction of *ChACBP1* Expression by Multiple Abiotic Stresses

**FIGURE 4:**
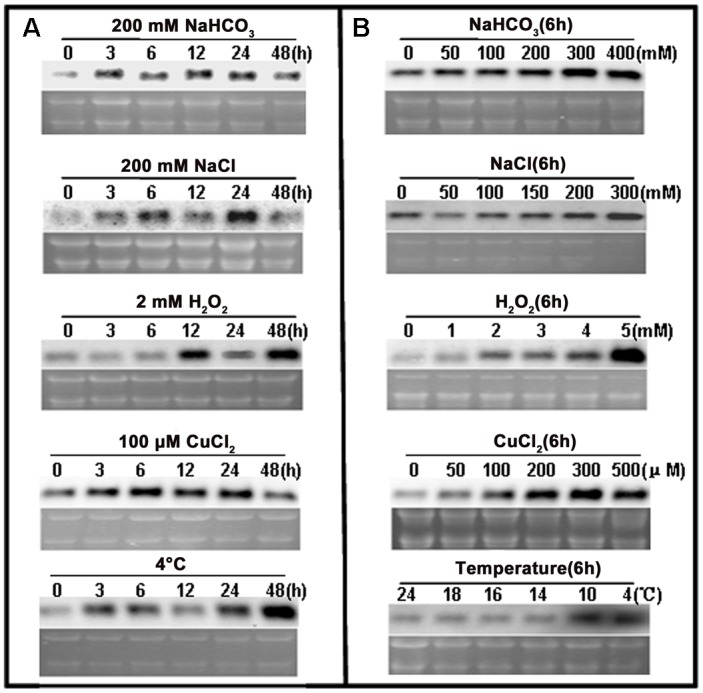
*ChACBP1* expression under abiotic stresses as detected by Northern blot analysis with digoxigenin-labeled *ChACBP1* cDNA probe. **(A)** Northern blot analysis using total RNA (3 μg) extracted from *Chlorella* cells treated with 200 mM NaHCO_3_, 200 mM NaCl, 2 mM H_2_O_2_, 100 μM CuCl_2_, or low temperature (4°C) at indicated time points. **(B)** Northern blot analysis using total RNA (3 μg) extracted from *Chlorella* cells treated with different concentrations of NaHCO_3_ (0, 50, 100, 200, 300, and 400 mM); NaCl (0, 50, 100, 150, 200, and 300 mM); H_2_O_2_ (0, 1, 2, 3, 4, and 5 mM); CuCl_2_ (0, 50, 100, 200, 300, and 500 μM); or different temperatures (24°C, 18°C, 16°C, 14°C, 10°C, and 4°C). All samples were collected at 6 h of treatment.

Northern blot analyses were used to examine the response of *ChACBP1* expression to various abiotic stresses. Analyses of total RNA extracted from SAT *Chlorella* exposed to NaHCO_3_, NaCl, H_2_O_2_, CuCl_2_, and 4°C for 0, 3, 6, 12, 24, and 48 h indicated that the *ChACBP1* mRNA levels increased in the 200 mM NaHCO_3_ treatment from 3 to 48 h, compared with the control (0 h). *ChACBP1* mRNA began to increase after 3 h in the 200 mM NaCl treatment and peaked after 24 h of treatment. *ChACBP* gene expression was induced at 12 h of the 2 mM H_2_O_2_ treatment and its expression level peaked at 48 h. The expression level of *ChACBP1* was higher after 3 h of the 100 μM CuCl_2_ treatment than in the control. Subsequently, its expression level increased gradually at 6, 12, and 24 h of the 100 μM CuCl_2_ treatment, but was similar to the control after 48 h. The expression level of *ChACBP1* began to increase after 3 h at 4°C and peaked at 48 h (Figure 4A). The level of *ChACBP1* expression was affected by the concentration as well as the duration of the stress elicitors.

Northern blot analyses were conducted using total RNA extracted from SAT *Chlorella* exposed to different concentrations of NaHCO_3_, NaCl, H_2_O_2_, and CuCl_2_, and different temperatures for 6 h. At this time point, *ChACBP1* mRNA levels were slightly higher in the 50 mM NaHCO_3_ treatment than in the control and were higher in the treatments with higher NaHCO_3_ concentrations. The peak expression level of *ChACBP1* among the NaHCO_3_ treatments was in the 300 mM NaHCO_3_ treatment. The highest *ChACBP1* expression level among the NaCl treatments was in the 300 mM NaCl treatment. The expression level of *ChACBP1* was slightly increased in the 1 mM H_2_O_2_ treatment, compared with the control, and its highest expression level among the H_2_O_2_ treatments was in the 5 mM H_2_O_2_ treatment. The expression of *ChACBP1* was induced by 50 μM CuCl_2_ and its expression level gradually increased as the concentration increased to 300 μM CuCl_2_. The expression levels of *ChACBP1* in the 18°C, 16°C, and 14°C treatments were not significantly different from that in the control (24°C), but were increased in the 10°C treatment to a level similar to that measured at 4°C (Figure 4B). These data suggested that the expression of *ChACBP1* was induced by NaHCO_3_, NaCl, H_2_O_2_, CuCl_2_, and low temperature stresses.

### ChACBP1 Localizes to the Cytosol

**FIGURE 5:**
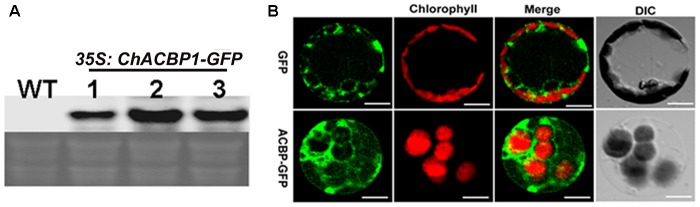
Subcellular localization of ChACBP1 in *Arabidopsis* protoplasts. **(A)** Northern blot analysis with *ChACBP1* cDNA probe in three independent pBI121-*ChACBP1*-GFP transgenic lines (lanes 1–3). WT, Wild type. **(B)** Confocal microscope images of protoplasts of *Arabidopsis* pBI121-*ChACBP1*-GFP line 2 indicating localization of ChACBP1-GFP in the cytosol. GFP empty vector is shown at the top. Bars = 20 μm.

The expression of *ChACBP1*-GFP mRNA in three independent pBI121-*ChACBP1*-GFP transgenic *Arabidopsis* lines was detected by Northern blot analysis using a *ChACBP1* cDNA probe (Figure 5A). Analyses of the shoots of line 2 expressing pBI121-*ChACBP1*-GFP revealed that the GFP signal was localized in the cytosol. The GFP control was also expressed in the cytosol (Figure 5B).

### Generation of *ChACBP1* Transgenic *Arabidopsis*

To test whether *ChACBP1* overexpression enhanced abiotic tolerance, the *ChACBP1* full-length cDNA was expressed in the *Arabidopsis* by an *Agrobacterium*-mediated method. Four independent *ChACBP1*-overexpressing lines (OX-1, OX-2, OX-3, and OX-4) were identified by Northern blot analysis using *ChACBP1* mRNA. A Southern blot analysis indicated that OX-2, OX-3, and OX-4 had one or two gene copies (Supplementary Figure S3).

### Overexpression of *ChACBP1* in *Arabidopsis* Enhanced Tolerance to Multiple Abiotic Stresses

**FIGURE 6:**
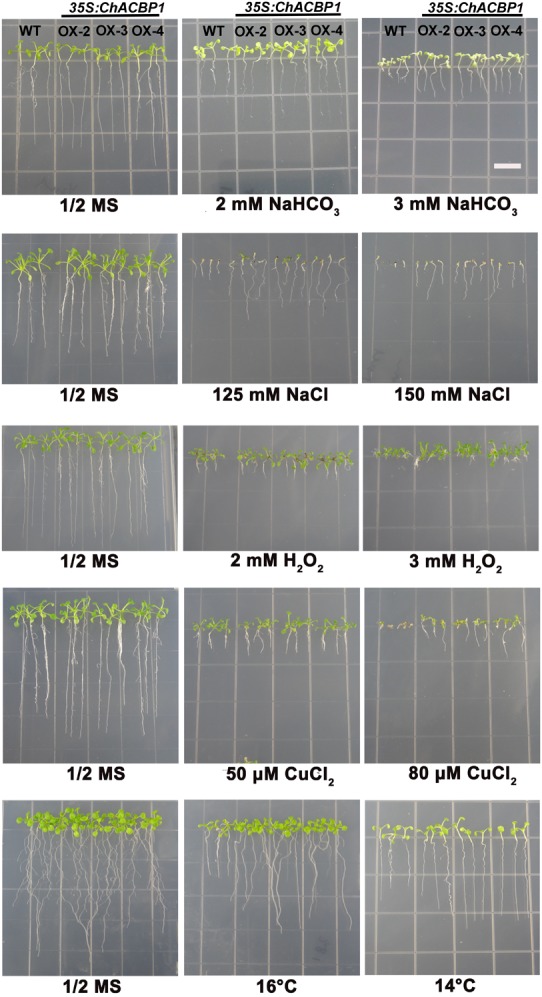
Effects of abiotic stresses on root length in wild-type (WT) and *ChACBP1-*overexpressing transgenic plants. Seeds of WT and transgenic lines were grown on 1/2 MS medium supplemented with 2 and 3 mM NaHCO_3_, 125 and 150 mM NaCl, 2 and 3 mM H_2_O_2_, or 50 and 80 μM CuCl_2_. Seedlings were grown for 7–14 days. For low temperature stress treatment, plants were grown at 16°C and 14°C for 30 days. Bars = 1 cm.

**FIGURE 7:**
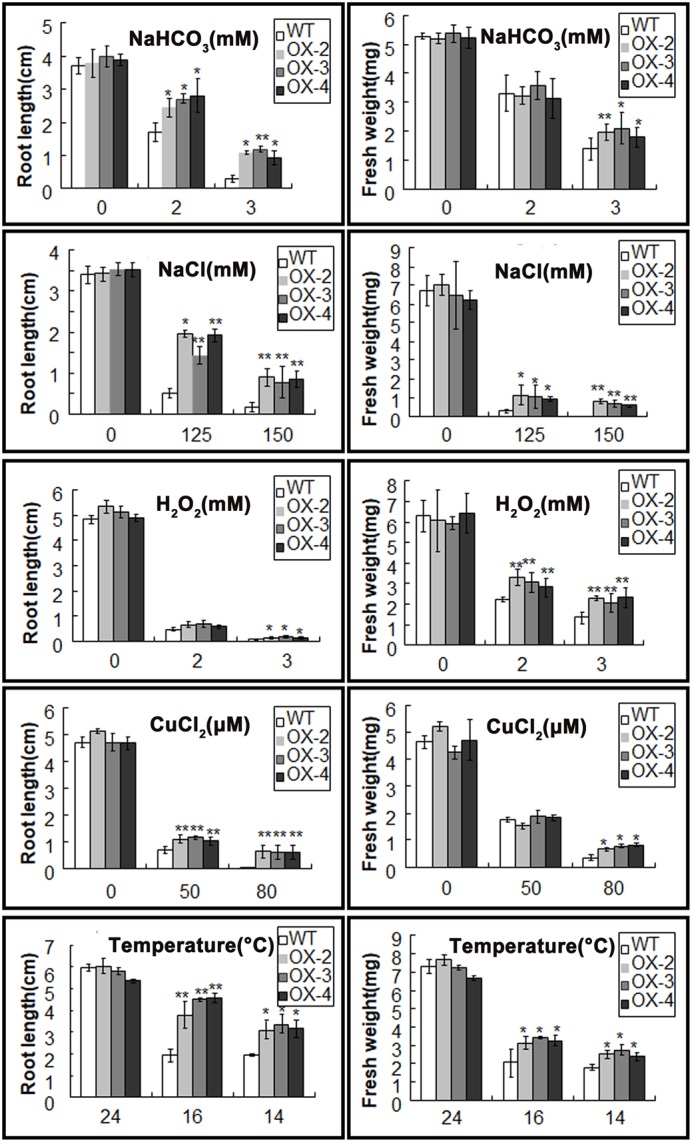
Root length and fresh weight of wild-type (WT) and *ChACBP1*-overexpressing transgenic plants after indicated stress treatments. Values are mean ± SE of four plants. Significant differences (*P* < 0.05) were determined by *t*-test. Asterisks indicate significant differences from WT (^∗∗^*P* < 0.01, ^∗^0.01 < *P* < 0.05).

When grown on 1/2 MS medium, the three transgenic lines (OX-2, OX-3, and OX-4) over-expressing *ChACBP1* were not noticeably different from WT (Figures 6, 7). Under 2 and 3 mM NaHCO_3_ treatment, root growth was significantly enhanced in *ChACBP1*-overexpressing *Arabidopsis* lines, compared with WT (*P* < 0.05). The entire seedling fresh weight was greater in the overexpressing lines than in the WT only in the 3 mM NaHCO_3_ treatment (*P* < 0.05; Figure 7). In the presence of 125 and 150 mM NaCl, the root length and fresh weight were higher in the overexpressing lines than in WT (*P* < 0.05; Figures 6, 7). When grown under oxidative stress (H_2_O_2_), root length was not notably different between the overexpressing lines and WT. However, the leaves of the overexpressing lines grew better than did those of WT (*P* < 0.05; Figures 6, 7). The fresh weights of the overexpressing lines were higher than those of WT in the 2 and 3 mM H_2_O_2_ treatments (*P* < 0.05; Figure 7). When the plants were exposed to CuCl_2_ and low temperature stresses, root lengths and fresh weights were higher in the overexpressing lines than in the WT (*P* < 0.05; Figures 6, 7).

**FIGURE 8:**
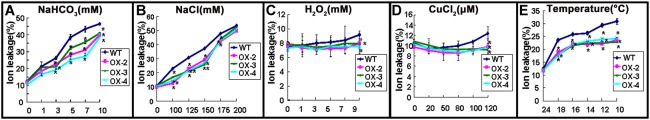
Electrolyte leakage of wild-type (WT) and *ChACBP1*-overexpressing (OX-2, OX-3, and OX-4) plants after 24 h of treatment with NaHCO_3_ (0, 1, 3, 5, 7, and 10 mM) **(A)**, NaCl (0, 100, 125, 150, 175, and 200 mM) **(B)**, H_2_O_2_ (0, 1, 3, 5, 7, and 9 mM) **(C)**, CuCl_2_ (0, 20, 50, 80, 100, and 120 μM) **(D)**, or low temperature (24°C, 18°C, 16°C, 14°C, 12°C, and 10°C) **(E)**. Values are means ± SD (*n* = 3) from three independent experiments. Asterisks indicate significant differences from WT (^∗∗^*P* < 0.01, ^∗^0.01 < *P* < 0.05).

To evaluate membrane injury after different stress treatments, electrolyte leakage was measured in 2-week-old seedlings from WT and *ChACBP1*-overexpressing plants treated with different concentrations of NaHCO_3_, NaCl, H_2_O_2_, and CuCl_2_, and different temperatures. The results showed that ionic leakage was not obviously different between WT and overexpressing lines in the 1 mM NaHCO_3_ treatment, but was significantly higher in overexpressing lines than in WT plants in the 3 to 10 mM NaHCO_3_ treatments (*P* < 0.05; Figure 8A). In comparison, the ionic leakage in *ChACBP1*-overexpressing seedlings was significantly lower (*P* < 0.05) than that in WT in the 100, 125, 150, and 175 mM NaCl treatments. There was no obvious difference in ionic leakage between WT and overexpression lines in the 200 mM NaCl treatment (Figure 8B). The ionic leakage was significantly lower (*P* < 0.05) in *ChACBP1*-overexpressing plants than in WT plants in all the temperature treatments (18°C, 16°C, 14°C, 12°C, and 10°C) (*P* < 0.05; Figure 8E). In the presence H_2_O_2_ and CuCl_2_, ionic leakage was lower in the overexpressing plants than in the WT only at the higher concentrations (9 mM H_2_O_2_ and 120 μM CuCl_2_, respectively) (*P* < 0.05; Figures 8C,D).

## Discussion

China has 99.13 million hectares of saline-alkali soil, which accounts for 10% of the world’s saline-alkali land. The alkalinity in these regions is generally high, resulting in oxidative and salt stress. Drought stress is also common in these environments. As a result, few plants can survive under such conditions. In recent years, the area of saline-alkali soil has gradually increased as a result of environmental changes and human activities (Wang et al., 2011; Qiao et al., 2015). These changes have resulted in severe losses in agricultural production. We previously reported that *Chlorella* JB6 can survive in extreme saline-alkali soil with pH > 9.5 (Qiao et al., 2015). Therefore, this species may be a useful resource for plant genetic engineering.

We isolated *ChACBP1* by screening a cDNA library of SAT *Chlorella* JB6. The full-length protein sequence was found to be homologous with the amino acid sequences of the small ACBPs from other species used to construct the NJ tree. Some functionally critical motifs in ACBPs have been relatively well conserved throughout evolution. However, there were some differences between the N-terminal or C-terminal regions of ChACBP1 and those of ACBPs in other species (Figure 2), suggesting that the function of ChACBP1 may be slightly different from that of ACBPs in other species. The YKQA and KWDAW motifs in the ACB conserved domain are considered to be essential for binding PC (Zhang et al., 2008). An evolutionary analysis of the *Chlorella, Arabidopsis*, and rice ACBP families indicated that ChACBP1 might be close to AtACBP6 and OsACBP4 in its structure and function. Therefore, the *ACBP* gene in *Chlorella* was considered to encode an acyl-CoA-binding protein. We tested the binding of ChACBP1 to phospholipids in filter-binding assays and observed that ChACBP1 bound PC (Figure 3), similar to the lipid-binding properties of AtACBP6 (Chen et al., 2008). It was hinted that ChACBP1 might transport multiple PCs associated with phospholipid metabolism. Phospholipids are the main components of cell membranes. In this study, the ion leakage (membrane stability index) of *ChACBP1*-overexpressing *Arabidopsis* was lower than that of WT under different stresses (Figure 8). This result suggested that ACBP may play a role in protecting the plasma membrane.

Our results showed that the *Chlorella* *ACBP1* gene was induced by NaHCO_3_, NaCl, H_2_O_2_, CuCl_2_, and low temperatures, and that overexpression of *ChACBP1* in yeast and *Arabidopsis* improved stress tolerance compared with that of their WT controls (Figures 1, 6). In cotton, *GhACBP3* and *GhACBP6* were found to be up-regulated under high salinity stress (Qin et al., 2016). In *Arabidopsis, ACBP2*-overexpressing lines showed enhanced tolerance to H_2_O_2_ (Gao et al., 2009), and both *AtACBP1*- and *AtACBP2*-overexpressors displayed enhanced tolerance to heavy metals (Pb or Cd) (Xiao et al., 2008; Gao et al., 2009). The expression level of *AtACBP6* was found to be up-regulated by cold treatment (Chen et al., 2008). In the present study, *ChACBP1* was isolated from a cDNA library expressed in yeast grown under NaHCO_3_ stress, and overexpression of *ChACBP1* in yeast and *Arabidopsis* improved their tolerance to NaHCO_3_ (Figures 1, 7). Because NaHCO_3_ treatment likely leads to other stresses, such as salinity, alkalinity, oxidative, and drought stresses, it is not surprising that ChACBP1 can confer tolerance to multiple abiotic stresses. However, the mechanism of ChACBP1 tolerance is still unclear.

**FIGURE 9:**
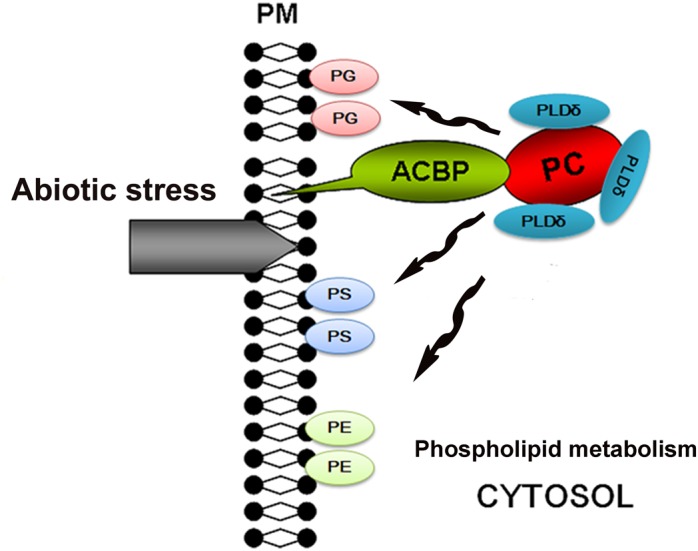
Hypothetical model for stress tolerance function of *Chlorella* ACBP. ACBP, acyl-CoA-binding protein; PC, phosphatidylcholine; PG, phosphatidylglycerol; PS, phosphatidyl-serine; PE, phosphatidylethanolamine; PLDδ, phospholipases Dδ; PM, plasma membrane.

Overexpression of *ACBP6* in *Arabidopsis* was shown to enhance the activities of phospholipase Dδ (PLDδ), which in turn enhanced cold tolerance by stabilizing the membrane skeleton (Chen et al., 2008). PLDδ can convert PC into PG, PS, and PE, all of which stabilize the cell membrane. Therefore, we propose a model in which exposure of the plasma membrane to abiotic stresses induces the expression of *ChACBP1*, and the subsequent increase in ChACBP1 protein leads to increased binding and transport of PC to the plasma membrane from within the cytosol. Hence, the increase in PG, PS, and PE contents increases the stability of the cell membrane (Figure 9). However, this model is still hypothetical. Like ACBPs from higher plants, ChACBP1 conferred tolerance to various abiotic stresses. The proposal that ChACBP1 mediates plant abiotic stress adaptation through phospholipid metabolism should be tested experimentally in further studies.

## Ethics Statement

This study was approved by the ethics committee of Zhejiang Agriculture and Forestry University.

## Author Contributions

SL and TT conceived and designed the research. KQ and MW conducted the experiments. KQ analyzed the data and wrote the manuscript. All authors revised and approved the manuscript.

## Conflict of Interest Statement

The authors declare that the research was conducted in the absence of any commercial or financial relationships that could be construed as a potential conflict of interest.

## Footnotes

1 http://www.ncbi.nlm.nih.gov

2 http://dnastar.com/t-products-lasergene.aspx
