# Supplementary material for: Overexpression of Acyl-CoA-Binding Protein 1 (ChACBP1) From Saline-Alkali-Tolerant Chlorella sp. Enhances Stress Tolerance in Arabidopsis
Source: Front Plant Sci. 2018 Nov 28;9:1772. doi: 10.3389/fpls.2018.01772 (PMC6282033; doi:10.3389/fpls.2018.01772)
Supplement: Supplementary file 1 [file Data_Sheet_1.docx]

**SUPPLEMENTARY MATERIAL**

**Overexpression of *acyl-CoA-binding protein 1 (ChACBP1)* from Saline-Alkali-Tolerant *Chlorella* sp. Enhances Stress Tolerance in *Arabidopsis***

Kun Qiao**^1,^** ^2^, Min Wang^3^, Tetsuo Takano^4^, Shenkui Liu**^1^**^*^

**^1^** The State Key Laboratory of Subtropical Silviculture, Zhejiang Agriculture and Forestry University, Lin’an, China

^2^ Shenzhen Key Laboratory of Marine Bioresource & Eco-environmental Science, Guangdong Engineering Research Center for Marine Algal Biotechnology, College of Life Science and Oceanography, Shenzhen University, Shenzhen, China

^3^ Key Laboratory of Saline-alkali Vegetation Ecology Restoration in Oil Field (SAVER), Ministry of Education, Alkali Soil Natural Environmental Science Center (ASNESC), Northeast Forestry University, Harbin 150040, China

^4^Asian Natural Environment Science Center (ANESC), University of Tokyo, Midori Cho 1-1-1, Nishitokyo City, Tokyo, 188-0002, Japan

* Corresponding author: Shenkui Liu

Email: [shenkuiliu@nefu.edu.cn](mailto:shenkuiliu@nefu.edu.cn)

Tel: (86)-0451-82191394

Fax: (86)-0451-82191394

This supporting information contains three figures.

Figures: Figure S1-S3

**Figures**


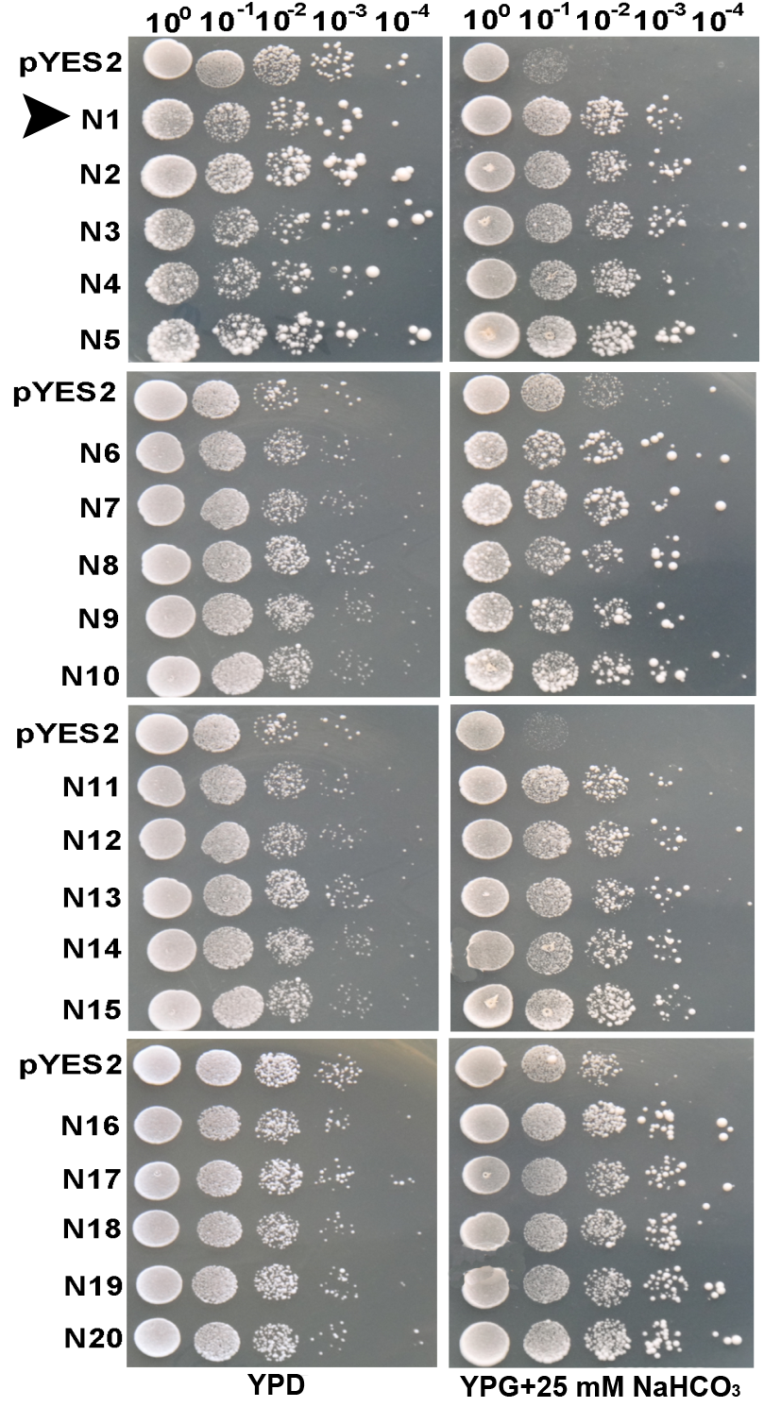


**Figure S1** Preparation of NaHCO_3_-tolerant overexpression yeast constructs.

Serial dilutions were spotted onto yeast extract/peptone/glucose media YPG agar plates supplemented with 25 mM NaHCO_3_ at the concentrations indicated. Solid yeast extract/peptone/glucose media (YPD) was the media control. Growth was monitored for 3-7 days at 30°C. Yeast cells containing the pYES2-ChACBP vector are indicated by a solid black arrow. Yeast cells containing pYES2 empty vector were used as controls.


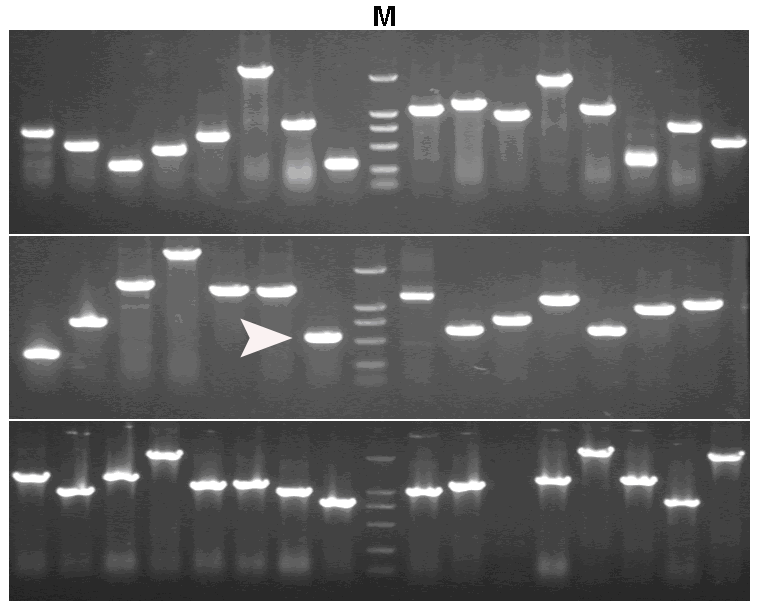


**Figure S2** Amplification of tolerant NaHCO_3_ gene candidates.

M: DL2000 (from top to bottom: 2000-bp, 1000-bp, 750-bp, 500-bp, 250-bp, and 100-bp). Tolerant yeast samples were screened, and DNA was extracted for sequencing. Presumed tolerant NaHCO_3_ genes were amplified using the pYES2 forward primer 5′-TTGATACCACTGCTTCTGCAGAATTCCAGCACACTG-3′ and the pYES2 reverse primer 5′-TCTCATCGTACCCCGATATCCATCACACTGGCGGC-3′. *ChACBP* gene is indicated by a solid white arrow.


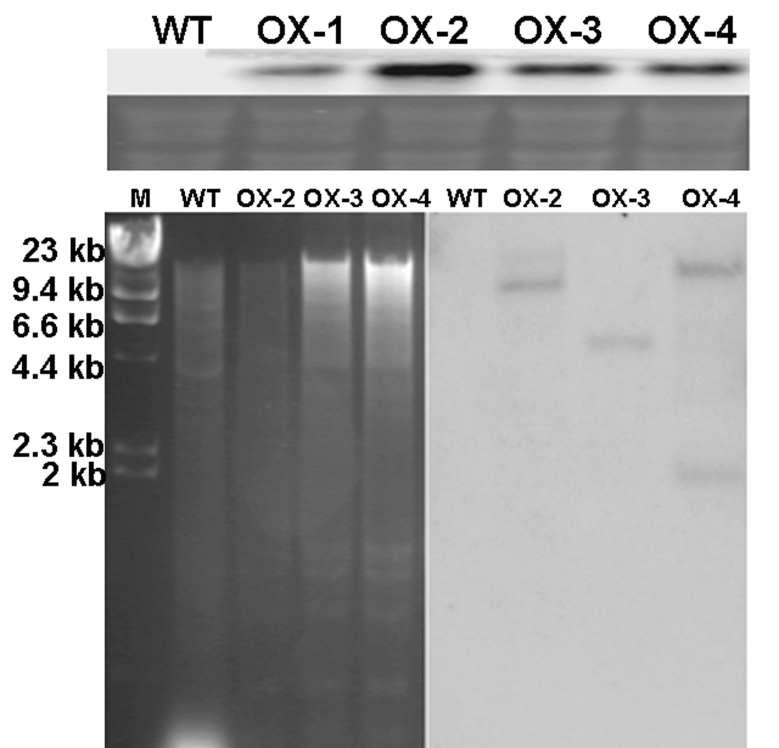


**Figure S3** Northern blot analysis of WT and *ChACBP1* overexpressing (OX-1, OX-2, OX-3, and OX-4) plants using *ChACBP* cDNA probe. *ChACBP1* overexpressing lines showed higher *ChACBP* expression than the wild type. Total RNA (3 μg) stained with ethidium bromide before blotting is shown at the bottom. Southern blot of wild type and *ChACBP1* overexpressing (OX-2, OX-3, and OX-4) plants using *ChACBP1* cDNA probe. *ChACBP1* overexpressing lines showed that OX-2 and OX-3 each had a single copy, while OX-4 had two copies. M: λDNA.
